# MTOR-Driven Metabolic Reprogramming Regulates *Legionella pneumophila* Intracellular Niche Homeostasis

**DOI:** 10.1371/journal.ppat.1006088

**Published:** 2016-12-12

**Authors:** Camille F. Abshire, Ana-Maria Dragoi, Craig R. Roy, Stanimir S. Ivanov

**Affiliations:** 1 Department of Medicine, Feist-Weiller Cancer Center, Louisiana State University Health Sciences Center - Shreveport, Shreveport, Louisiana, United States of America; 2 Department of Microbial Pathogenesis, Yale University School of Medicine, New Haven, Connecticut, United States of America; 3 Department of Microbiology and Immunology, Louisiana State University Health Sciences Center - Shreveport, Shreveport, Louisiana, United States of America; DUMC, UNITED STATES

## Abstract

Vacuolar bacterial pathogens are sheltered within unique membrane-bound organelles that expand over time to support bacterial replication. These compartments sequester bacterial molecules away from host cytosolic immunosurveillance pathways that induce antimicrobial responses. The mechanisms by which the human pulmonary pathogen *Legionella pneumophila* maintains niche homeostasis are poorly understood. We uncovered that the *Legionella*-containing vacuole (LCV) required a sustained supply of host lipids during expansion. Lipids shortage resulted in LCV rupture and initiation of a host cell death response, whereas excess of host lipids increased LCVs size and housing capacity. We found that lipids uptake from serum and *de novo* lipogenesis are distinct redundant supply mechanisms for membrane biogenesis in *Legionella*-infected macrophages. During infection, the metabolic checkpoint kinase Mechanistic Target of Rapamycin (MTOR) controlled lipogenesis through the Serum Response Element Binding Protein 1 and 2 (SREBP1/2) transcription factors. In *Legionella-*infected macrophages a host-driven response that required the Toll-like receptors (TLRs) adaptor protein Myeloid differentiation primary response gene 88 (Myd88) dampened MTOR signaling which in turn destabilized LCVs under serum starvation. Inactivation of the host MTOR-suppression pathway revealed that *L*. *pneumophila* sustained MTOR signaling throughout its intracellular infection cycle by a process that required the upstream regulator Phosphatidylinositol-4,5-bisphosphate 3-kinase (PI3K) and one or more Dot/Icm effector proteins. *Legionella*-sustained MTOR signaling facilitated LCV expansion and inhibition of the PI3K-MTOR-SREPB1/2 axis through pharmacological or genetic interference or by activation of the host MTOR-suppression response destabilized expanding LCVs, which in turn triggered cell death of infected macrophages. Our work identified a host metabolic requirement for LCV homeostasis and demonstrated that *L*. *pneumophila* has evolved to manipulate MTOR-dependent lipogenesis for optimal intracellular replication.

## Author Summary

To evade host defenses certain bacterial pathogens establish unique membrane-bound compartments within infected cells where they replicate to large numbers. The membrane integrity of these vacuolar compartments is crucial for pathogen survival because rupture initiate host anti-microbial defenses. The causative agent of Legionnaires’ disease, *Legionella pneumophila* is a prototypical intracellular bacterial pathogen that establishes and replicates within a vacuolar compartment. How *Legionella* ensures integrity of the vacuolar compartment as it expands to accommodate hundreds of bacteria is unknown. We found that *Legionella* subverts the metabolism of its host by activating the central metabolic checkpoint kinase MTOR to sustain membrane production needed for the expansion of its intracellular niche. MTOR activation favored *Legionella* replication by increasing the housing capacity of the *Legionella*-occupied vacuole whereas interference with MTOR function led to destabilization, premature rupture and activation of host anti-microbial responses. Our work provides novel insight into how pathogens maximize intracellular replication by exploiting the host metabolic processes.

## Introduction

Residence within a membrane-bound organelle is a survival strategy common to intracellular bacterial pathogens [1–3]. *Legionella pneumophila*, the etiological agent of a severe pneumonia known as Legionnaires’ disease, can establish a unique endoplasmic reticulum (ER)-derived vacuole in amoebae and also in mammalian macrophages [4–8]. The molecular strategies that *Legionella* has evolved to survive within a broad range of protozoan hosts allow the bacterium to replicate in alveolar macrophages during human infections [9]. The kinetics and the mechanism of LCV biogenesis are largely conserved in different host cells [4]. Within 30 min of phagocytosis, *Legionella* blocks endocytic maturation and initiates phagosomal membrane remodeling through the recruitment and fusion with early secretory vesicles [6,10–14]. By 4 hrs post-infection the LCV fuses with the ER and an intracellular niche that supports bacterial replication is established; however, the LCV retains features distinct form the ER [15], such as accumulation of ubiquitinated proteins [13,16]. In synchronized infections, bacterial replication starts at ~ 4 hrs and by 16 hrs a single LCV expands to and contains hundreds of bacteria [16,17]. At the end of a single replication cycle, the number of bacteria per vacuole varies widely among established LCVs [18,19], however the underlying mechanisms that support such heterogeneity are unclear. Thus, it is important to identify the processes that favor and the processes that limit bacterial replication within established LCVs.

*Legionella* species encode a type IVb secretion system (T4bSS), known as the Dot/Icm apparatus, which translocates over 300 bacterial effector proteins directly into the host cytosol [20–22]. The T4bSS is required for intracellular survival and deletion mutants lacking single structural components of the Dot/Icm apparatus are avirulent because they fail to block endocytic maturation [23–25]. Collectively, the Dot/Icm effector proteins facilitate niche biogenesis and homeostasis [20,22]. One example is the SdhA effector, which maintains LCV integrity by counteracting, through an unknown mechanism, the activity of the secreted *Legionella* phospholipase PlaA [18]. In macrophage infections, vacuoles containing Δ*sdhA* mutants rupture during the early stages of bacterial replication, release bacterial products in the host cytosol and trigger pyroptosis—an inflammatory host cell death that restricts bacterial replication [18,26,27]. Mutants lacking LidA, another Dot/Icm effector, also establish a rupture-prone LCV and fail to grow intracellularly, but only when LidA is deleted in combination of either WipB or MavP [28]. The evolution of multiple bacterial regulators highlights the importance of an intact LCV membrane for *Legionella* intracellular survival. Host regulators of LCVs stability are unknown; however, membrane biogenesis regulators likely are involved because in the course of 12 hrs the size of the pathogen-containing vacuole expands.

Membrane biogenesis in eukaryotes occurs at the ER, which is the main site of *de novo* synthesis of phopholipids and cholesterol from metabolic precursors [29]. Adaptive lipogenesis is mainly regulated through increase in gene expression of lipogenic enzymes mediated by the SREPB1/2 transcription factors [30–32]. In turn, SREBPs are controlled by the master metabolic checkpoint serine/threonine kinase MTOR [33–36]. MTOR responds to cues from energy and nutrient sensing pathways to initiate a global anabolic state in eukaryotic cells when conditions are favorable [37,38]. MTOR nucleates two distinct protein complexes—TORC1 and TORC2 that have unique as well as shared components, distinct substrate specificities and subcellular localizations [39,40]. Raptor and Rictor are unique scaffolding proteins that define the TORC1 and TORC2 complex, respectively [38,41]. Several pharmacological inhibitors of MTOR have been identified, including the ATP-competitive MTOR inhibitors PP242 and Torin1/2 as well as the macrolide rapamycin that inhibits phosphorylation of only a subset of TORC1 substrates [42–45]. In cell cultures, MTOR inhibitors block *de novo* lipogenesis and *in vivo* genetic models attribute lipogenic functions to both TORC complexes [34,36,37].

In addition to lipogenesis, MTOR controls immunity to infection by repressing inflammation and autophagy [46–49]. In epithelial cells infected by *Salmonella* and *Shigella*, an amino acid starvation response blocks MTOR signaling to promote autophagy [50]. Infections of primary BMMs with *Legionella* encoding a functional Dot/Icm system triggers a host-driven suppression of MTOR through ubiquitination-induced degradation of the upstream MTOR regulator Akt that promote inflammation [51]. In this study, we found that host-driven MTOR suppression requires Myd88 and interferes with a *Legionella*-induced MTOR-dependent lipogenic reprogramming mediated by one or more Dot/Icm effector proteins that control LCV homeostasis.

## Results

### Loss of the TLR adaptor Myd88 reveals TLR-independent MTOR signaling elicited by Dot/Icm+ *Legionella*

Previously, we reported that TLR-dependent activation of MTOR is suppressed when macrophages become infected with *Legionella* encoding a functional Dot/Icm system [51]. Thus, the role of the TLR adaptor Myd88, which is required for LPS-induced MTOR signaling [46,48], was investigated in the context of *Legionella* infection. To eliminate rapid pyroptosis induced by *Legionella* flagellin, flagellin-deletion mutants *(ΔflaA*) were used in this study [52]. Also, the cells were serum-starved for 10hrs prior to and for the duration of the infections to avoid growth factor-induced MTOR activation. There was no detectible decrease in cell viability caused by serum withdrawal under these conditions.

**Fig 1:**
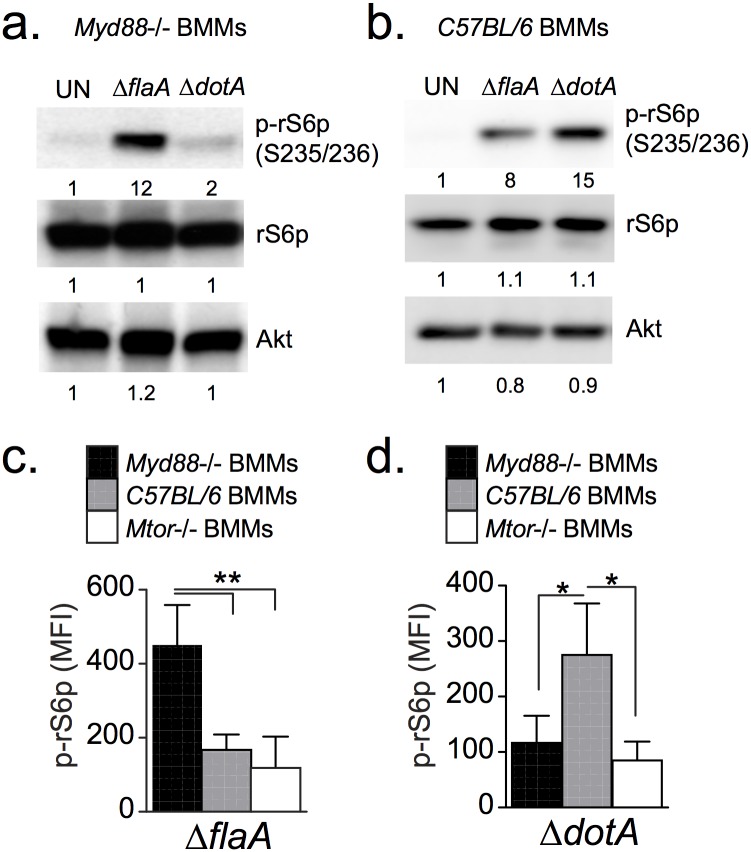
Analysis of rS6p phosphorylation in bone marrow-derived macrophages. Immunoblot analysis of cell lysates from *Myd88*^-/-^ **(a)** or C57BL/6 **(b)** BMMs that were serum-starved and infected with *Legionella* (MOI = 20) for 4 hrs as indicated under serum-free conditions. Quantified band intensities are normalized to uninfected conditions (UN) and listed below each blot. Single-cell analysis of rS6p phosphorylation in serum-starved BMMs infected with either *ΔflaA* **(c)** of *ΔdotA* **(d)**. Graph shows means and 95% confidence intervals of phospho-rS6p fluorescent intensity signal for at least 100 infected cells for each condition. * p<0.05, ** p<0.005 (one-way ANOVA). **(a-d)** A representative of three biological replicates is shown for each experiment.

To assess mTOR signaling, *Myd88*^-/-^ BMMs were infected with a pathogenic Dot/Icm+ strain (Δ*flaA*) or an avirulent Dot/Icm- strain (*ΔdotA*) and phosphorylation of the ribosomal S6 protein (rS6p) was measured by immunoblot analysis to assess MTOR activity (Fig 1a). Phosphorylation of rS6p at serine residues 235 and 236 by the S6 kinase 1 (S6K1) was used as readout for mTOR signaling because S6K1 is directly activated by TORC1 [53]. As expected, a *ΔdotA* strain did not induce rS6p phosphorylation in *Myd88*^-/-^ BMMs because TLR signaling is defective; surprisingly, the Dot/Icm+ strain triggered a robust response (Fig 1a). These data are in sharp contrast with the attenuated rS6p phosphorylation elicited by the Δ*flaA* strain as compared to the Δ*dotA* strain in BMMs derived from C57BL/6 mice (Fig 1b). To analyze MTOR activity specifically in infected cells we quantitated phospho-rS6p fluorescence intensity using single-cell immunofluorescence analysis. These studies confirmed that robust phospho-rS6p was elicited by a Δ*flaA* strain in *Myd88*^-/-^ BMMs and by a Δ*dotA* strain in C57BL/6 BMMs (Fig 1c and 1d and S1 Fig). Taken together, these data demonstrate that MTOR suppression in response to virulent *Legionella* requires MyD88 signaling, and that in the absence of MyD88 signaling virulent *Legionella* augment MTOR activation.

**Fig 2:**
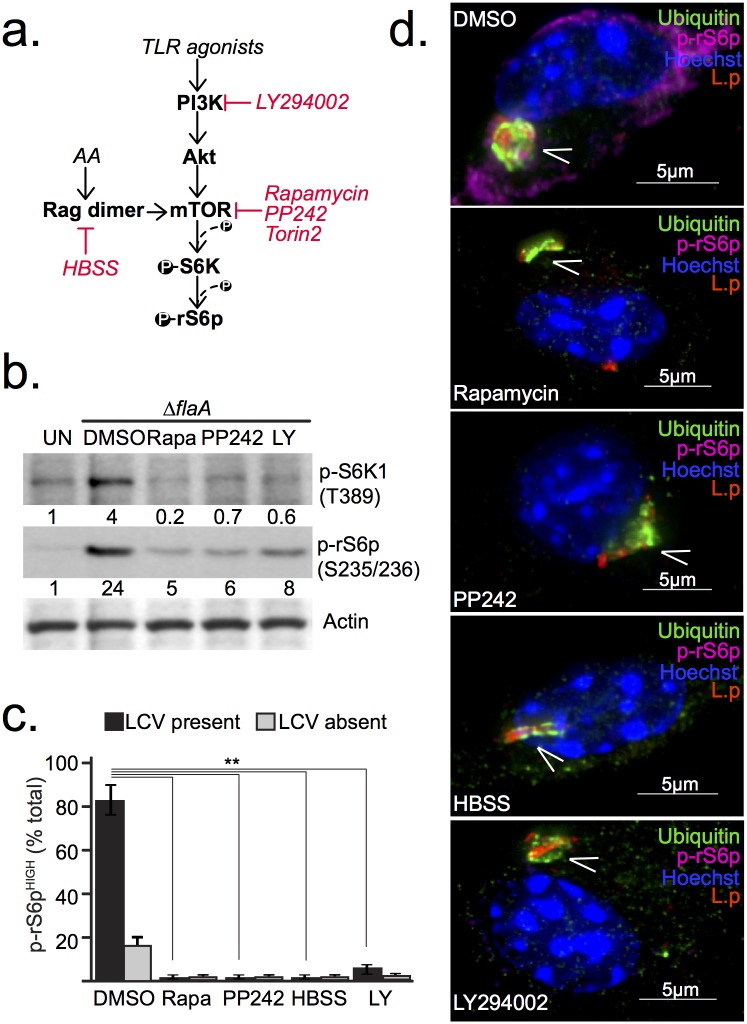
*Legionella*-induced phosphorylation of rS6p requires MTOR and PI3K activity. **(a)** Simplified schematics of the PI3K/MTOR signaling axis indicating the inhibitors used in this study. PI3K is inhibited by LY294002. Rapamycin, PP242 and Torin2 act on MTOR and Hanks' Balanced Salt Solution (HBSS) blocks MTOR by starving cells for amino acids. **(b-d)** Analyses of serum-starved *Myd88*^-/-^ BMMs unstimulated or infected with *ΔflaA* (MOI = 20) for 5hrs under serum-free conditions. Inhibitors—Rapamycin (200nM), PP242 (5μM), LY294002 (10μM)—or HBSS were added at the time of infection synchronization at 60 min post infection. **(b)** Immunoblot analysis of S6K1 and rS6p phosphorylation from cell lysates showing quantified band intensities normalized to uninfected conditions (UN). **(c)** Single cell immunofluorescence analysis of phospho-rS6 positive (MFI>300) *Myd88*^-/-^macrophages exposed to *ΔflaA* (MOI = 20). Graphed are the means and standard deviations (s.d) of technical triplicates for the two distinct groups within the cell population—infected (LCV present) and uninfected (LCV absent) for each condition. At least 100 cells were analyzed for each condition. ** p<0.005 (one-way ANOVA) **(d)** Immunofluorescense micrographs of representative infected cells from each condition stained with anti-*L*. *pneumophila* (L.p), anti-p-rS6p (S235/236), anti-ubiquitinated proteins (FK2) antibodies and Hoechst 33342. Arrowheads indicate *Legionella*-containing vacuoles, Bar = 5μm. **(b-d)** A representative of three biological replicates is shown for each experiment.

To exclude the possibility of an MTOR-independent phospho-rS6p response to the Δ*flaA* strain in *Myd88*^-/-^ BMMs, MTOR function was inhibited in infected cells through various approaches (Fig 2a). Addition of the MTOR inhibitors PP242 or rapamycin to synchronized *Legionella* infections of *Myd88*^-/-^ BMMs reduced phosphorylation of S6K1 and its substrate rS6p (Fig 2b). Single cells microscopy confirmed that phospho-rS6p signal was diminished in infected macrophages when MTOR inhibitors were added (Fig 2c and 2d). Amino acid starvation (HBSS treatment) or PI3K inhibition also ablated *Legionella*-induced phospho-rS6p in *Myd88*^-/-^ BMMs (Fig 2b–2d). Under these infection conditions, MTOR inhibition was initiated after the infections were synchronized and therefore did not interfere with bacterial uptake. LCV maturation and bacterial viability were also unaffected by MTOR inhibition as evidenced by the ubiquitin accumulation on vacuoles containing bacteria with normal morphology (Fig 2d). Thus, loss of phospho-rS6p under MTOR inhibiting conditions was not a result of bacterial killing by the host cell or perturbations in LCV biogenesis. Together, these data indicate that phosphorylation of rS6p induced by *Legionella* in *Myd88*^-/-^ BMMs requires PI3K/MTOR signaling and is an indicator of MTOR activity in the cell.

### MTOR activation by *Legionella* is a Dot/Icm effector-driven process that is independent of intracellular niche biogenesis

**Fig 3:**
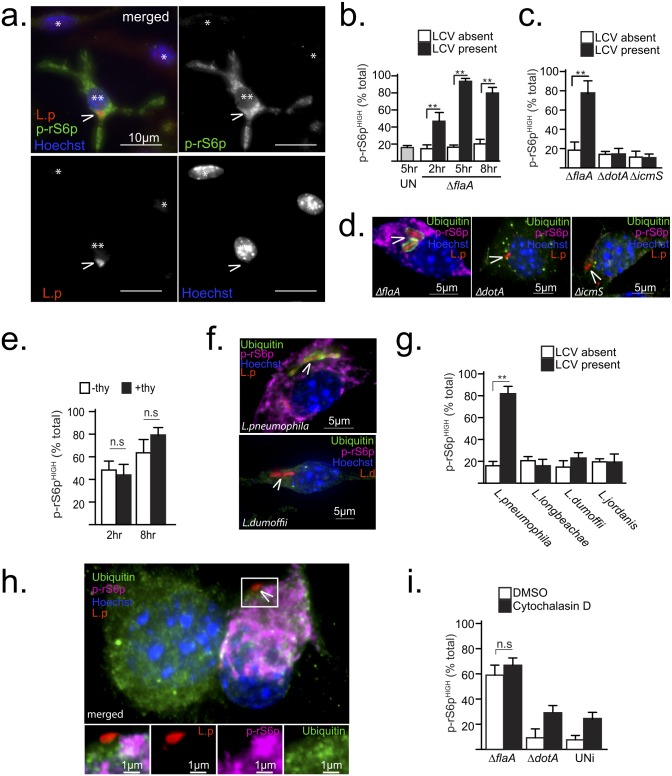
*Legionella*-induced MTOR activation is a Dot/Icm effector-driven process independent of intracellular niche biogenesis. **(a-h)** Show results from synchronized infections (MOI = 20) under serum-free conditions of serum-starved *Myd88*^-/-^ BMMs treated as indicated. **(a)** Immonofluorescent micrograph of *ΔflaA*-infected (**) or neighboring uninfected (*) cells at 6 hrs post-infection stained with anti-*L*. *pneumophila* (L.p), anti-p-rS6p (S235/236) antibodies and Hoechst 33342. Arrowheads indicate *Legionella*-containing vacuoles, Bar = 10μm. **(b-c** and **g)** Single cell immunofluorescence analysis of phospho-rS6 positive (MFI>300) BMMs exposed to various *Legionella* strains for the indicated times **(b)** or for 8hrs **(c** and **g)**. Means ± s.d of technical triplicates for the two distinct groups within the cell population—infected (LCV present) and uninfected (LCV absent) for each condition are shown. **(d)** Micrographs of cells infected with the indicated strains for 8hrs and stained with anti-*L*. *pneumophila* (L.p), anti-p-rS6p (S235/236), anti-ubiquitinated proteins (FK2) antibodies and Hoechst 33342. Arrowheads indicate intracellular bacteria, Bar = 5μm. **(e)** Quantitation of p-rS6p positive cells (MFI>300) in synchronized infections with a *thyA ΔflaA* strain in the presence (+thy) or absence (-thy) of thymidine for the indicated time periods. Means ± s.d of technical triplicates are shown. **(f-g)** Synchronized macrophages infections with different *Legionella* species are shown. **(f)** Representative micrographs of cells infected with *L*. *pneumophila* or *L*. *dumoffii* for 8hrs (MOI = 20) and stained as in **(d)**, which are quantitated in **(g)**. **(h-i)** Cells were treated with cytochalasin D (5μM) or vehicle (DMSO) for 15 min prior to and for the duration of infections (3hrs) with the indicated bacterial strains. **(i)** Quantitation of p-rS6p positive cells (MFI>300) in contact with bacteria (*ΔflaA* and *ΔdotA*) or uninfected cells (UNi). Means ± s.d of technical triplicates are shown. **(h)** Representative micrograph of macrophages treated with cytochalasin D (5μM) and infected with *ΔflaA* and stained as in **(d)**. Arrowhead indicates a bacterium in contact with the macrophage, Bar = 1μm. At least 50 cells **(i)** or 100 cells **(b, c, e** and **g)** were analyzed for each condition. A representative of two **(f-h)** or three **(a-g)** biological replicates is shown for each experiment. **(b, c, e, g** and **i)** n.s—not significant, ** p<0.005 (unpaired T-test).

To gain insight into the mechanism by which *Legionella* infections activate MTOR in *Myd88*^-/-^ BMMs, the kinetics of MTOR signaling was analyzed using fluorescence microscopy by quantification of phospho-rS6p positive cells at different times post-infection (Fig 3a and 3b). In synchronized infections, at 2hrs post-infection over 40% of infected cells stained positive for phospho-rS6p as compared to only 15% of neighboring uninfected cells (Fig 3b). In a population of unstimulated serum-starved BMMs only 17% of the cells were phospho-rS6p positive (Fig 3b). Over time, the number of phospho-rS6p positive infected cells further increased to over 80% at 5hrs and 8hrs post-infection. In contrast, the number of phospho-rS6p positive uninfected cells remained at basal level. Thus, a significant number of infected cells activated MTOR prior to initiation of bacterial replication and sustained high MTOR activity throughout the *Legionella* intracellular lifecycle.

Because a functional Dot/Icm apparatus is essential for MTOR activation by *Legionella* (Fig 1a), a strain lacking the chaperone IcmS, which loads effectors into the Dot/Icm apparatus [54,55], was used to determine whether the apparatus or secretion of effectors through the apparatus is important for MTOR activation. The *ΔicmS* bacteria assemble a functional Dot/Icm apparatus but exhibits a severe defect in translocation of most effectors and therefore fails to establish an intracellular niche that supports bacterial replication in macrophages [56]. Both *ΔdotA* and Δ*icmS* strains failed to induce MTOR activity (Fig 3c and 3d) indicating that signaling is unlikely a host response to the Dot/Icm apparatus but rather efficient translocation of effectors is necessary for MTOR activation. Such a requirement indicates that Dot/Icm effectors mediate MTOR activation directly or indirectly through LCV biogenesis.

A potential indirect trigger for MTOR activation could be the need of the infected cell to offset a metabolic demand placed by a rapidly expanding membrane-bound organelle that contains replicating bacteria. Such a mechanism would require bacterial replication; however, a thymidine auxotroph strain activated MTOR irrespective of thymidine presence, indicating that organelle expansion is not the trigger (Fig 3e). Next, MTOR activation by other *Legionella* species was examined. *L*. *dumoffii*, *L*. *longbeachae* and *L*. *jordanis* have conserved LCV biogenesis programs but considerable diversity in their Dot/Icm effector repertoires. All the tested *Legionella* species established LCVs that supported bacterial replication, but only *L*. *pneumophila* infections activated MTOR (Fig 3f and 3g). In co-infections of *Myd88*^-/-^ BMMs, *L*. *dumoffii* failed to repress MTOR signaling induced by *L*. *pneumophila* indicating that *L*. *dumoffii* neither induces nor suppresses MTOR (S2 Fig). Thus, MTOR is activated by a *L*. *pneumophila*-specific process and is independent of LCV expansion.

Next, the actin polymerization inhibitor cytochalasin D was used to investigate the possibility that LCV establishment could lead to MTOR induction. Treatment with cytochalasin D blocks LCV formation because phagocytosis is inhibited and the bacteria are retained on the extracellular interface of the plasma membrane. However, cell-surface associated bacteria still inject Dot/Icm effectors in the host cytosol [57]. Macrophages in contact with Δ*flaA* bacteria, but not *ΔdotA* bacteria, triggered MTOR irrespective of cytochalasin D treatment (Fig 3h and 3i). Therefore, MTOR induction by *L*. *pneumophila* is independent of LCV biogenesis altogether and strongly support the existence of Dot/Icm effector(s) that promote PI3K-dependent MTOR signaling. The pentuple deletion mutant lacking 71 Dot/Icm effector genes [58] still activated MTOR (S2c Fig), therefore a forward genetic screen would be required to identify the *Legionella* MTOR regulator(s).

### Blocking *Legionella*-induced MTOR activation triggers host cell death

**Fig 4:**
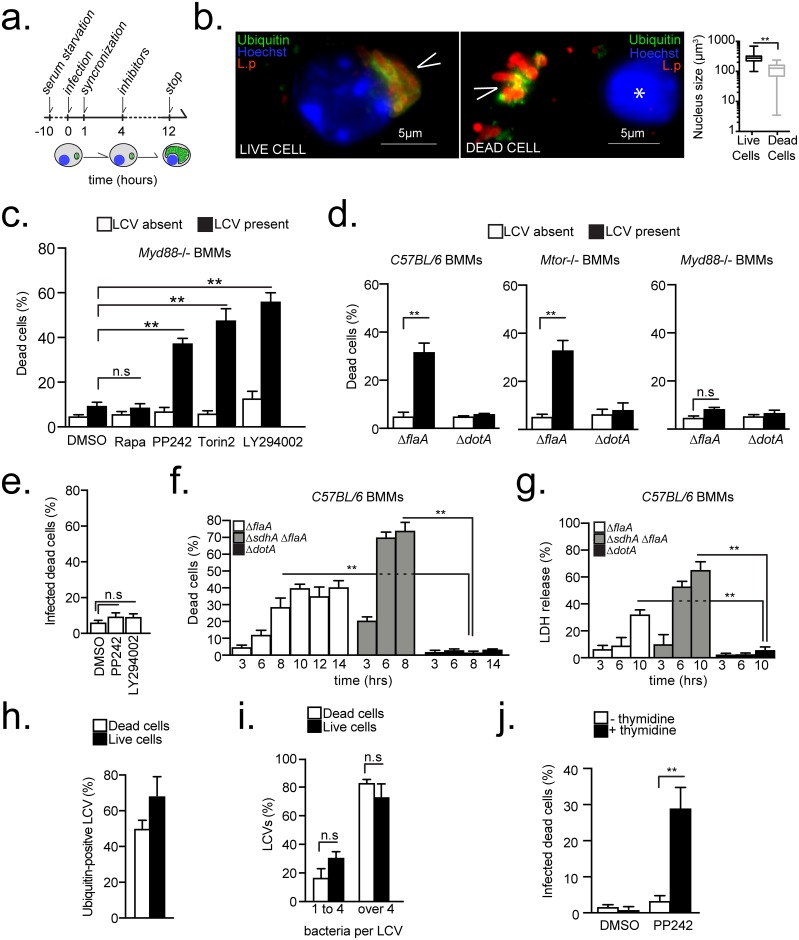
Loss of MTOR function triggers a host cell death response that requires bacterial replication. **(a)** Experimental scheme for the results shown in **(b-e and g-j)**. **(b-j)** BMMs were serum-starved and infected under serum-free conditions either at MOI = 20 **(b-e and g-j)** or MOI = 10 **(f)**. **(b)** Micrographs showing representative of live and dead macrophages infected with *Legionella* for 12 hrs and stained with anti-*L*. *pneumophila* (L.p), anti-ubiquitinated proteins (FK2) antibodies and Hoechst 33342. Arrowheads indicate LCVs, Bar = 5μm. (*) marks the condensed nucleus of the dead cells. The mean nuclear volumes ± s.d of at least 100 live and 100 dead cells are graphed. **(c-d)** Quantitation of infected and neighboring uninfected *Myd88*^-/-^ **(c-d)**, C57BL/6 **(d)** and *Mtor*^-/-^ **(d)** macrophages with condensed nuclei after infections with *ΔflaA* **(c-d)** or Δ*dotA* **(d)**. Means ± s.d of technical replicates of dead cell as percentage of total cells in each condition are shown. **(e)** Quantitation of infected *Myd88*^-/-^ BMMs with condensed nuclei after infection with *L*. *dumoffii* and treatment with inhibitors or vehicle as indicated. **(f-g)** Kinetics of the cell death response in C57BL/6 BMMs under serum starvation conditions infected as indicated. Quantitation of infected cells with condensed nuclei **(f)** and LDH released in the culture supernatants **(g)** are shown. **(h-i)** Analyses of ubiquitin recruitment **(h)** and LCV size **(i)** in live and dead *Myd88*^-/-^ BMMs infected with *ΔflaA* and treated with PP242. **(j)** Cell death in infected *Myd88*^-/-^ BMMs with *thyA ΔflaA* strain treated with vehicle (DMSO) or PP242 in the presence or absence of thymidine. **(c, e, h-j)** Rapamycin (250nM), PP242 (2.5μM), LY294002 (10μM), Torin2 (300nM). **(c-j)** Means ± s.d of technical triplicates for each condition are shown. At least 50 cells **(e, h-j)** or 200 cells **(c-d)** were analyzed for each condition. A representative of two **(e-j)** or three **(c-d)** biological replicates is shown for each experiment. **(b-j)** n.s—not significant, ** p<0.005 (unpaired T-test).

Next, the impact of sustained MTOR activation on *Legionella* intracellular life cycle was determined in macrophage infections under serum-free conditions that were allowed to progress for less than a single infection cycle (~16hrs) in the presence or absence of MTOR inhibitors (Fig 4a). Inhibitors were added at 4 hrs post infection and timed to coincide with the start of bacterial replication to eliminate potential interference with bacterial uptake and trafficking. A morphologically distinct population of infected cells characterized by condensed nuclei—decreased nuclear volume (from 284 to 141μm^3^) and increased fluorescence when stained with the DNA dye Hoechst 33342—was detected clearly under all infection conditions (Fig 4b and S3 Fig). Aberrant condensed nuclear morphology is a prototypical hallmark used to identify dying cell [59].

The number of dead *Myd88*^-/-^ BMMs infected with *Legionella*, identified by their aberrant nuclear morphology, significantly increased from 8% to over 40% when PI3K (LY294002) or MTOR (PP242, Torin2) were inhibited (Fig 4c). This cell-death phenotype was not a result of inhibitor-induced cell toxicity because neighboring uninfected cells were not affected. Interestingly, rapamycin, which inhibits the phosphorylation of a subset of TORC1 substrates, such as rS6p [45], did not induce cell death in infected or uninfected cells (Fig 4c). Taken together, these data demonstrate that suppression of the *Legionella*-induced PI3K/MTOR signaling triggers a cell death response in infected *Myd88*^-/-^ BMMs through a rapamycin-insensitive mechanism.

If *Legionella*-induced MTOR activity interferes with a host cell death response, it would be predicted that in the presence of TLR-signaling the host Akt/MTOR-ubiquitination pathway will phenocopy the effect of the pharmacological inhibitors in *Myd88*^-/-^ BMMs infections. Indeed, a significant increase in the number of dead *Legionella*-infected cells was observed at 12 hrs post-infection in macrophages derived from C57BL/6 mice (Fig 4d). Similar results were obtained when macrophages derived from mice with MTOR ablation in the myeloid compartment were infected (Fig 4d). Importantly, under serum-free conditions, neighboring uninfected bystander macrophages from infections with either the Δ*flaA* or the *ΔdotA* strain did not undergo cell death. Also, *Myd88*^-/-^ macrophages, which sustain MTOR activity, remained alive when infected with the Δ*flaA* strain. Thus, the host Akt/MTOR-ubiquitination pathway as well as pharmacological or genetic ablation of MTOR function triggered a cell-death response specifically in macrophages infected with virulent *Legionella* encoding functionally intact Dot/Icm system. However, the Dot/Icm apparatus itself did not trigger this cell death response because infections with *L*. *dumoffii*, which encodes a homologous Dot/Icm system, did not induce host cell death in *Myd88*^-/-^ BMMs even when MTOR inhibitors were present (Fig 4e).

### The host cell death in *Legionella*-infected macrophages triggered by MTOR suppression requires bacterial replication

Multiple bacteria within ubiquitin-positive LCVs were observed frequently within macrophages undergoing MTOR suppression-dependent cell death (similar to Fig 4b); therefore, the kinetics of the cell death response was investigated. Under MTOR suppressing conditions, BMMs with aberrant nuclear morphology infected with Δ*flaA* bacteria increased gradually starting at 6hrs after bacterial replication is initiated and peaked at 10-14hrs post-infection (Fig 4f). The release in the culture supernatants of the cytosolic enzyme lactate dehydrogenase (LDH) by dying macrophages also increased with similar kinetics (Fig 4g). In contrast, infections with the Δ*sdhA* mutant strain, which triggers cell death prior to bacterial replication [18], produced distinct kinetics that peaked at 6hrs post-infection (Fig 4f and 4g). Moreover, majority of LCVs within dead macrophages in MTOR-suppressing conditions were mature ubiquitin-positive LCVs that have supported bacterial replication (i.e. contained more than 4 bacteria) (Fig 4h and 4i). Thus, bacterial replication is likely initiated prior to host cell death. To determine whether bacterial replication is required for initiation of the MTOR-regulated host cell death, *Myd88*^-/-^ BMMs were infected with a thymidine auxotroph Δ*flaA* strain. In those infections, host cell death was detected only in the presence of PP242 and thymidine, demonstrating that bacterial replication together with MTOR suppression are required for induction of host cell death (Fig 4j).

### Serum-derived lipids rescue the host cell death phenotype brought by MTOR inhibition

**Fig 5:**
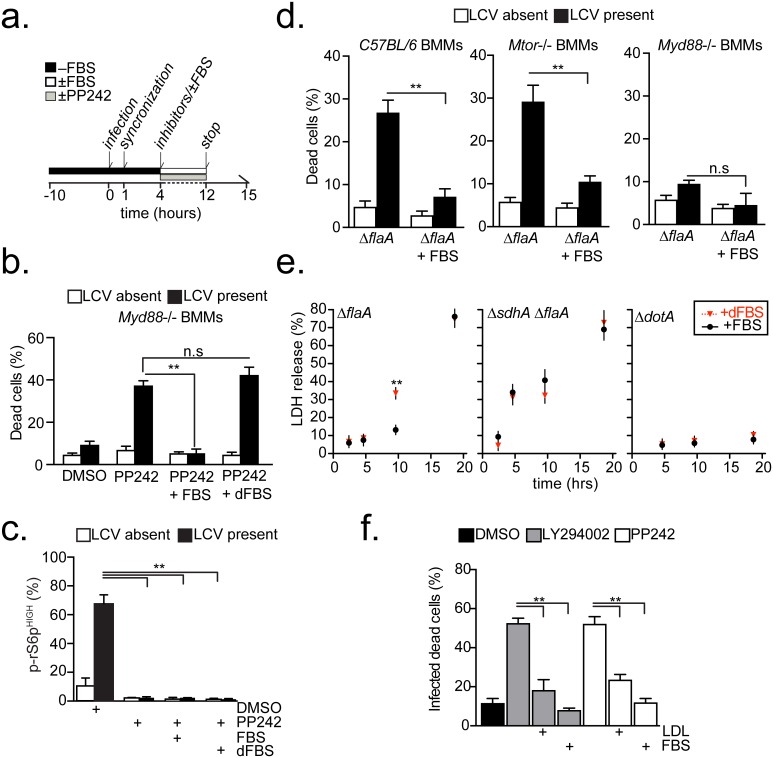
*Legionella*-dependent cell death triggered by MTOR suppression is blocked by lipids supplementation. **(a)** Experimental scheme for the results shown in **(b-d, f)**. **(b-c)** *Myd88*^-/-^ BMMs with condensed nuclei **(b)** or positive for phospho-rS6p (MFI>300) **(c)** produced by infections with *ΔflaA* (MOI = 20) and the indicated treatments. Infected and neighboring uninfected cells were quantified for each category. **(d)** Infected and neighboring uninfected BMMs with condensed nuclei after infections with *ΔflaA* or *ΔdotA* (MOI = 20) in the presence/absence of FBS. **(e)** Kinetics of LDH release by C57BL/6 BMMs infected as indicated (MOI = 10) in the presence of FBS or dFBS. **(f)** Infected *Myd88*^-/-^ BMMs with condensed nuclei produced by *ΔflaA* infection (MOI = 20) and the indicated treatments. **(b-f)** PP242 (2.5μM), LY294002 (10μM), FBS (10%), dFBS (10%), human LDL (10mg/ml). **(b-f)** Means ± s.d of technical triplicates for each condition are shown. At least 50 cells **(c,f)** or 100 cells **(b,d)** were analyzed for each condition. A representative of two **(e-f)** or three **(b-d)** biological replicates is shown for each experiment. **(b-f)** n.s—not significant, ** p<0.005 (unpaired T-test).

The detection of a *Legionella*-induced host cell death response in C57BL/6 BMMs under serum starvation is intriguing because it is well established that *ΔflaA L*. *pneumophila* replicates in these macrophages without initiating a cell death response in the presence of fetal bovine serum (FBS) [52]. Therefore, the possibility that FBS could account for the distinct phenotypes was investigated by infecting macrophages for 12hrs (less than a single replication cycle) with *Legionella* in the presence/absence of FBS and MTOR inhibitors (Fig 5a). Under these conditions, serum supplementation effectively blocked host cell death in *Legionella*-infected *Myd88*^-/-^ BMMs treated with the MTOR inhibitor PP242 (Fig 5b). Because, FBS did not interfere with PP242 activity (Fig 5c), these data indicate that FBS compensated for the loss of MTOR function. Supplementation with FBS also blocked the *Legionella*-induced cell death in BMMs derived from C57BL/6 and from *Mtor*^-/-^ mice (Fig 5d) providing further evidence that FBS compensates for the reduction in MTOR function brought by multiple MTOR-suppression mechanisms.

Although MTOR is a global metabolic regulator, the potential loss of MTOR-dependent lipogenesis was investigated first because: (1) FBS can compensate for loss of MTOR-driven lipogenesis and when FBS is available cells default to lipid uptake over *de novo* synthesis to reduce energy expenditure [29]; (2) serum is a major source for cellular lipids and lipid-precursors in vertebrates [29]; (3) rapamycin failed to trigger cell death in *Legionella* infections and MTOR-driven lipogenesis is insensitive to rapamycin in some cell types [36,60,61]. The master lipogenic transcription factors SREBP1 and SREBP2 promote lipogenesis downstream of MTOR by increasing gene expression of lipid uptake regulators and biosynthesis enzymes [33,34,62]. Consistent with induction of a lipogenic transcriptional response, a significant increase in expression of *Srebf1* as well as several of its target genes was detected in *Myd88*^-/-^ BMMs infected with Δ*flaA* but not Δ*dotA Legionella* (S4 Fig). Therefore, a functional Dot/Icm apparatus is critical for initiation of MTOR signaling and the induction of a pro-lipogenic transcriptional program in *Legionella*-infected macrophages (S4 Fig).

If serum-derived lipids prevented host cell death in infected cells by compensating for the loss of MTOR-driven lipogenesis, the phenotype should be sensitive to depletion of serum lipids. Thus, the cell death response brought by MTOR-inhibition was investigated in the presence of delipidated FBS (dFBS) (cholesterol 2mg/dL, triglycerides 59mg/dL) or complete FBS (cholesterol 34mg/dL, triglycerides 61mg/dL) (Fig 5). Delipidated FBS failed to block the *Legionella*-induced host cell death brought by PP242-treatment in *Myd88*^*-/-*^ macrophages (Fig 5b) or by the host MTOR-suppression pathway (Fig 5d and 5e), demonstrating that FBS-derived lipids compensate for the lost MTOR function in infected macrophages. Furthermore, addition of purified Low-density lipoprotein (LDL) particles—the main cholesterol transport carriers in serum—was sufficient to block cell death in *Legionella*-infected *Myd88*^-/-^ BMMs triggered by LY294002 (PI3K inhibitor) or PP242 (MTOR inhibitor) (Fig 5f).

Next, the *Legionella*-induced MTOR-regulated cell death response and its dependency on lipids starvation was investigated in human macrophages. *Legionella*-infected phorbol-ester differentiated U937 macrophage cells were treated with PP242 in the presence of FBS or dFBS. Similar to the results with mouse BMMs, the number of dead infected U937 increased only when PP242 was added under serum-free conditions or in the presence of delipidated FBS; addition of PP242 and lipids-containing FBS blocked the PP242-induced cell death (S5 Fig). Collectively, these data link MTOR-driven lipogenesis with the *Legionella*-induced cells death response and indicate that MTOR activation or presence of exogenous lipids prevents a premature death of infected macrophages.

### MTOR maintains LCV integrity through activation of *de novo* lipogenesis

Due to the unique features of the *Legionella*-induced cell death: (1) requirement for bacterial replication and (2) loss of host lipogenesis, it was investigated whether insufficient supply of lipids could increase membrane tension and lead to LCV rupture during expansion. Elegant infection studies with a *ΔsdhA* strain, which resides in a rupture-prone LCV, have demonstrated that loss of LCV integrity releases bacterial molecules in the host cytosol leading to flagellin-independent cell death of infected macrophages [18].

**Fig 6:**
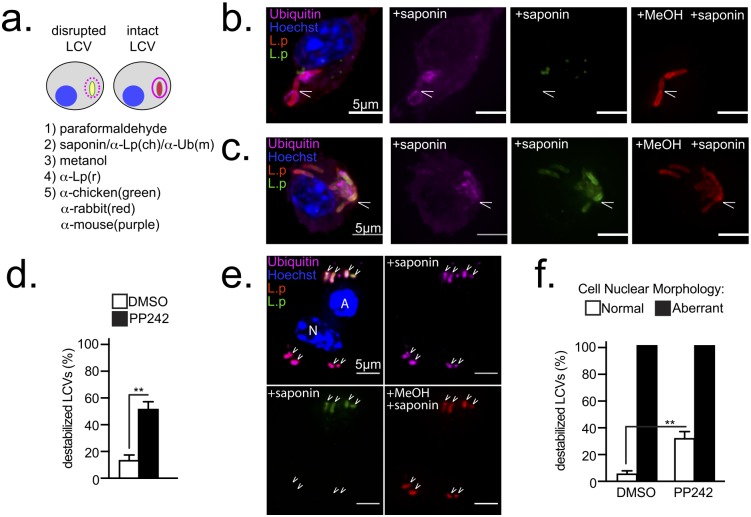
MTOR inhibition destabilizes LCVs. **(a)** Schematic for detection of leaky LCVs by selective plasma membrane permeabilization of infected cells. Single-positive bacteria reside in stable LCVs; double-positive bacteria reside in disrupted LCVs **(b-f)** Serum-starved *Myd88*^-/-^ macrophages infected with *ΔflaA L*. *pneumophila* (MOI = 20) for 7hrs. PP242 was added at the time of synchronization– 60min p.i. **(b-c)** Micrographs of representative stable **(b)** or leaky **(c)** LCVs are shown. Cells were stained as indicated in **(a)**. Arrowheads indicate LCVs. Bar = 5μm **(d)** Quantitation of destabilized LCVs in cells treated as indicated. **(e)** Micrographs of representative destabilized LCVs (double-positive) in BMM with aberrant (A) nuclear morphology and stable LCVs (single-positive) in a neighboring cell with normal (N) nuclear morphology Bar = 5μm **(f)** Quantitation of destabilized LCVs in BMMs with normal or aberrant nuclear morphology. BMMs treated as indicated. **(b-f)** PP242 (2.5μM). **(d-f)** Means ± s.d of technical triplicates for each condition are shown. At least 100 LCVs were analyzed for each condition. **(b-f)** A representative of three biological replicates is shown. **(d** and **f)** ** p<0.005 (unpaired T-test).

To discriminate between intact and leaky LCVs, the vacuole ability to prevent cytosolic anti-*Legionella* antibody from accessing the LCV lumen was investigated. First, the plasma membrane was selectively permeabilized with the amphipathic glycoside saponin and cells were stained with anti-*Legionella* chicken antibody as well as anti-ubiquitin antibody. Next, all membrane compartments were permeabilized with methanol and cells were stained with anti-*Legionella* rabbit antibody (Fig 6a). Thus, bacteria within intact LCVs are detected only after methanol incubation and remain single positive, whereas bacteria in leaky LCVs are accessible after saponin treatment and become double positive (Fig 6b and 6c). Detection of the ubiquitinated proteins that accumulate on the cytosolic interface of the LCV membrane during infection was used to control for saponin activity. In *Myd88*^-/-^ BMMs infected with the Δ*flaA* strain for 7hrs, 16% of the LCVs were leaky as indicated by detection of the vacuole-residing bacteria after saponin treatment (Fig 6c and 6d). The number of leaky LCVs increased to 51% following PP242 treatment indicating that MTOR suppression compromises LCV integrity. Although leaky LCVs were detected in BMMs with normal nuclear morphology (Fig 6c), all LCVs harbored by cells with aberrant nuclear morphology were leaky (Fig 6e and 6f). These data indicate that MTOR suppression destabilizes LCVs.

**Fig 7:**
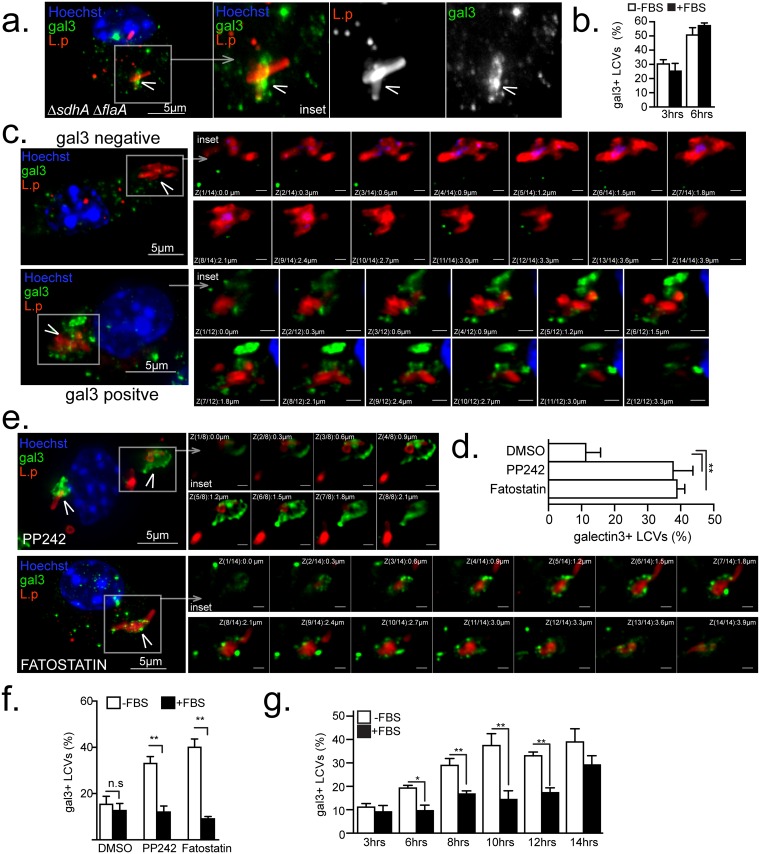
Serum lipids, SREPB1/2 and MTOR regulate LCV stability. Serum-starved *Myd88*^-/-^ **(a-f)** or C57BL/6 **(g)** BMMs or were infected by the indicated strains (MOI = 20) in synchronized infections for 12 hrs **(a-f)** or for the indicated time periods **(g)** in the absence **(a-g)** or presence **(f-g)** of FBS. **(a-b)** Galectin 3 accumulation onto LCVs harboring *ΔsdhA ΔflaA*. **(a)** projection micrograph of a representative infected cell with the inset showing the LCV **(b)** Kinetic analysis of Galectin 3+ LCVs harboring *ΔsdhA ΔflaA*. **(c,e)** Representative projection micrographs of Galectin 3 positive **(c,e)** or negative **(c)** LCVs harboring Δ*flaA* after treatments with inhibitors **(e)** or vehicle alone **(c)**. The insets show all individual planes of the projection image. Quantitation of Galectin 3+ LCVs after the indicated treatments in the absence **(d** and **f)** or presence **(f)** of FBS. **(g)** Kinetics of emergence of Galectin 3 positive LCV under serum starvation or replete conditions. **(c-g)** PP242 (2.5μM), fatostatin (4μM), FBS (10%). **(b,d,f** and **g)** Means ± s.d of technical triplicates for each condition are shown. At least 100 LCVs were analyzed for each condition. A representative of two **(b, f-g)** or three **(a, c-e)** biological replicates is shown for each experiment. **(b, d, f** and **g)** n.s—not significant, ** p<0.005 (unpaired T-test) **(a, c** and **e)** Cells were stained with anti-galectin3, anti-*Legionella* antibodies and Hoechst 33342. Arrowheads indicate the LCVs. Bar = 5μm.

To measure LCV rupture the recruitment of the cytosolic lectin Galectin-3 was assessed. Galectin-3 binds to lumenally occluded glycoprotein epitopes and accumulates onto membrane-bound organelles only when their epitopes become exposed upon membrane disruption [18,63–65]. Unstable LCVs, such as the ones harboring the *ΔsdhA* mutant, become Galectin-3 positive upon rupture (Fig 7a and 7b) [18]. Galactin-3 preferentially accumulates at the sites of membrane rupture [18,66] thus produces a wide-variety of non-uniform straining pattern onto the LCVs (S6a and S6b Fig). Accumulation of Galectin-3 onto LCVs within infected *Myd88*^*-/-*^ BMMs following MTOR inhibition was investigated. At 12 hrs post infection ~13% of the LCVs in vehicle-control treated cell were decorated with Galactin-3 (Fig 7c and 7d) and treatment with PP242 increased the number of Galactin-3+ LCVs to over 36% (Fig 7d and 7e). However, this number is likely an underestimate because Galactin3 staining was undetectable in dead macrophages and PP242-treatment increased the number of infected cells with condensed nuclei under serum-free conditions. MTOR inhibition did not cause global destabilization of pathogen-containing organelles because the number of Galactin-3+ LCVs harboring *L*. *dumoffii* did not increase after PP242 treatment (S6c and S6d Fig). In addition, MTOR inhibitors did not affect *L*. *pneumophila* replication and viability *in vitro* (S7 Fig), thus LCV rupture is unlikely a consequence of an interrupted LCV biogenesis program brought by an inhibitor-induced toxic effect on the residing bacteria.

Inhibition of lipogenesis at a step downstream of MTOR with the SREPB1/2 peptidomimetic inhibitor fatostatin, which specifically prevents the release of SREBP1/2 from the ER [67], destabilized LCV similar to PP242 treatment (Fig 7d and 7e). Importantly, addition of FBS restored LCV integrity even in the presence of PP242 and fatostatin as evidenced by the decreased number of Galactin-3+ LCVs (Fig 7f). Under MTOR suppressing condition, Galactin-3+ LCVs increased after bacterial replication was initiated and the kinetics of Galactin-3 acquisition by LCVs harboring *ΔflaA* bacteria mirrored the kinetics of host cell death response (Figs 7g vs. 4f and 4g). These data demonstrate that LCV membrane integrity during expansion is maintained through a continuous supply of lipids, either through uptake or *de novo* synthesis. Thus, interference with MTOR-driven lipogenesis results in LCV rupture during expansion and initiation of host cell death likely through the release of bacterial products in the host cytosol. Unlike MTOR suppression-induced instability, rupture of LCVs harboring Δ*sdhA* bacteria preceded bacterial replication and was not rescued by FBS supplementation indicating that MTOR and SdhA stabilize the LCVs via distinct mechanisms (Fig 7b).

### Lipids supply determines the housing capacity of the *Legionella* intracellular niche

Because the lipids supply/demand ratio is important for LCV homeostasis, its effects on the housing capacity of the pathogen-containing vacuole were investigated. In *Legionella*-infected cells, the LCV membrane contours the residing bacteria [15] and therefore the volume of the bacterial mass in the vacuole is proportional to the volume of the LCV membrane. In previous studies, housing capacity of a LCV was determined by manually counting the residing bacteria; however, the accuracy and reliability of this approach deteriorates for vacuoles containing more than 12 bacteria, which forces investigators to group large LCVs in a single category. To overcome this obstacle, an unbiased computer-based 3D image analysis was utilized to enumerate accurately bacteria within vacuoles independent of LCV size and topology. This analysis measures the volume of the bacterial mass within a LCV from reconstructed Z-stacks (0.3μm planes distance) of infected cells. To image the whole cell, cell height determined the Z-stack depth (4.5μm to 13μm). Next, the number of bacteria that can fit within the volume of the bacterial mass was extrapolated mathematically based on an algorithm derived from volume measurements of LCVs in which the bacteria can be counted manually (S8a–S8c Fig). When the algorithm was back tested it exhibited excellent correlation between counted and calculated number of bacteria per cell (*R*^*2*^ = 0.9287, p = 2.11X10^-38^) (S8d Fig). This automated volume-to-bacteria conversion approach facilitates reliable enumeration of bacteria even in large LCVs containing over a hundred bacteria (S8e and S8f Fig and S1 Video). Most 3D-imaging systems capable of volume measurements of binary masks should be able to enumerate intracellular bacteria using this methodology.

**Fig 8:**
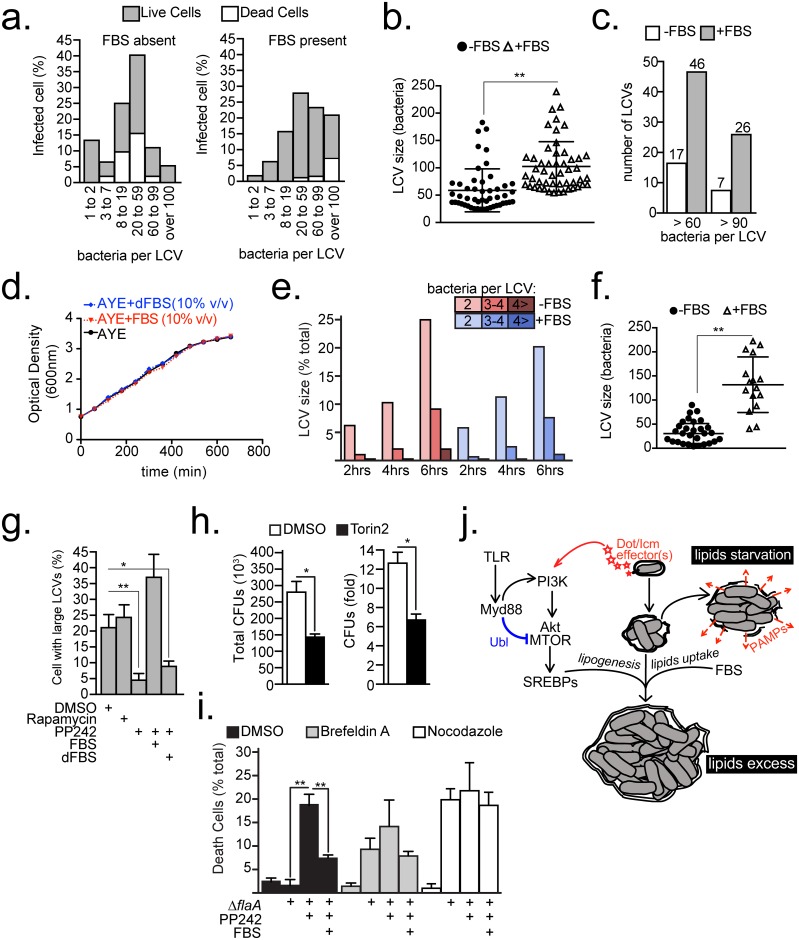
Host lipids dictate the LCV housing capacity. **(a-c, f)** Size analysis of LCVs harbored by C57BL/6 BMMs infected with *ΔflaA* bacteria (MOI = 20) for 15 hrs after 60 min synchronization. Cells were serum-starved prior to infection for 10hrs. LCV sizes were measured through 3D microscopy analysis of infected cells as detailed in S8 Fig. **(a)** Relative distributions of sizes of LCV harbored by live and dead cells produced by infections in the presence/absence of FBS. Live/dead distinction was determined morphologically by nuclear condensation. **(b-c)** Size analysis of the 50 largest LCVs produced by Δ*flaA* infections shown in **(a)**. **(d)** *Legionella* growth in axenic cultures supplemented with FBS or delipidated FBS (dFBS) (10% v/v). **(e)** Kinetic analysis of LCVs that support bacterial replication in C57BL/6 BMMs infected with *ΔflaA* bacteria. FBS was added or omitted after the infection synchronization at 30min post infection. At least 200 LCVs were scored for each condition. **(f)** Size analysis of LCVs harbored by cells with condensed nucleus from **(a)**. **(g)** Percentage of *Myd88*^-/-^ BMMs harboring large LCVs (bacteria>20) produced by 12 hrs synchronized infections with Δ*flaA* bacteria. Cell treatments were initiated at 4 hrs post infection as indicated. **(h)** *L*. *pneumophila* intracellular growth in *Acanthamoeba castellanii* over 48hrs in the presence of DMSO or Torin2 (300 nM), MOI = 5. **(i)** Percentage of *Myd88*^-/-^ BMMs with condensed nuclei uninfected or infected with *ΔflaA* bacteria for 9hrs. Infections were synchronized at 60min and various treatments were added at 6hrs as indicated. **(j)** Model for MTOR-dependent regulation of LCV homeostasis through the lipogenesis and serum-derived lipids. Abbreviations: ubiquitin ligase (UBL), pathogen-associated molecular patterns (PAMPs) **(b, f-g, h-i)** Means ± s.d of technical triplicates for each condition are shown. **(a** and **g)** At least 100 LCVs were analyzed for each condition. A representative of two **(d, h-i)** or three **(a-c, e-g)** biological replicates is shown for each experiment. **(a, g** and **h)** PP242 (2.5 μM), Brefeldin A (17.8 μM), Nocodazole (20 μM), FBS (10% v/v), dFBS (10% v/v) **(b, f-i)** * p<0.05, ** p<0.005 (unpaired T-test).

The relationship between lipid supply and LCV size was investigated in C57BL/6 BMMs, which suppress MTOR when infected by *Legionella*. In this system, exogenous FBS restores the lipids supply lost due to MTOR suppression. Thus, LCVs sizes were measured at the end of a single *Legionella* intracellular replication cycle (~16 hrs) in the presence/absence of FBS (similar to Fig 5a). In synchronized infections, the LCV sizes formed a Gaussian distribution with a peak at the 20–59 bacteria per LCV size bin irrespective of FBS supplementation (Fig 8a). However, FBS addition increased the population of large LCVs containing more than 60 bacteria (Fig 8a). Comparison between the fifty Iargest LCVs detected in each condition, revealed that FBS increased the average number of bacteria per LCV (from 58 to 102) and the number of LCVs containing more than 60 bacteria (from 17 to 46) (Fig 8b and 8c). FBS did not increase bacterial growth in axenic cultures (Fig 8d). Moreover, during the early stages of intracellular replication the number of bacteria per LCV remained similar in the presence or absence of FBS (Fig 8e) indicating that the LCV size increase observed in the later stages of infection was not a result of accelerated or prematurely initiated bacterial replication but likely due to LCV membrane biogenesis.

Next, the size of LCVs harbored by cells with aberrant nuclear morphology was analyzed because host cell death caused by vacuole rupture is rapid and likely captures the critical inflection point of cellular lipid-exhaustion when supply becomes insufficient. In the presence of FBS, infected dead cells harbored on average 132 bacteria and most of the LCVs exceeded 100 bacteria whereas under serum-free conditions the average size was reduced to 32 bacteria and very large LCVs (bacteria>100) were not detected in the population of dead cells (Fig 8a and 8f). Under serum-free conditions, when membrane biogenesis depends on lipogenesis, the size distribution curve of LCVs harbored by dead cells was broad, indicating that the threshold for LCV rupture varied (Fig 8a and 8f). Conversely, in the presence of FBS, most of the LCVs harbored by dead macrophages were very large, consistent with delayed LCV rupture and prolonged bacterial replication when lipids supply to infected cells is sustained (Fig 8a and 8f).

To determine if *Legionella*-induced MTOR-dependent lipogenesis similarly prolongs intracellular replication, the number of large LCVs harbored by *Myd88*^-/-^ BMMs infected for 12 hrs (as in Fig 5a) in the presence/absence of mTOR inhibitors were analyzed. Inhibition of MTOR with PP242, but not rapamycin, decreased the number of large LCVs consistent with regulation of LCV expansion and stability by *Legionella*-induced MTOR signaling (Fig 8g). Importantly, addition of FBS, but not delipidated FBS, sustained the number of large LCVs in the presence of PP242. Therefore, lipogenesis and lipid uptake are important functionally redundant determinants of the LCV housing capacity in *Legionella*-infected cells.

However, some large LCVs remained intact even under lipid-starvation conditions (Fig 8a and 8g), suggesting additional MTOR-independent mechanism can facilitate LCV expansion. Functional redundancy is a well-documented survival strategy by *Legionella* that facilitates a broad host range [28,58]. Indeed, MTOR inhibition with TORIN2 moderately reduced *L*. *pneumophila* intracellular replication in the protozoan host *Acanthamoeba castellanii* supporting MTOR-dependent and -independent LCV expansion mechanisms (Fig 8h). One potential MTOR-independent LCV expansion mechanism could be a direct membrane acquisition from other intracellular compartments. Indeed, early secretory vesicles tether and directly fuse with the LCV [6,68] thus it was tested whether the secretory compartment contributes to LCV expansion during MTOR suppression. To this end, *Myd88-/-* BMMs infected with Δ*flaA* bacteria were treated for 3hrs with PP242 and brefeldin A to disperse the Golgi compartment or nocodazole to block microtubules-dependent vasicular trafficking during the late LCV expansion phase (Fig 8i). Neither inhibitor treatment potentiated the PP242-induced cell death indicating that it is unlikely that the secretory compartment directly contributes membranes for the late LCV expansion.

Taken together, our data support a model in which an MTOR-dependent and an MTOR-independent pathway regulate LCV expansion by *Legionella* species. In the MTOR-dependent pathway, *Legionella pneumophila* manipulates MTOR function through secreted Dot/Icm effector(s) to drive lipogenesis and facilitate maximal niche expansion (Fig 8j). Insufficient host lipogenesis during *Legionella pneumophila* intracellular replication results in premature exhaustion of cellular lipids leading to loss of LCV membrane integrity, release of bacterial products in the cytosol of infected cells and cell death. However, serum-derived lipids can compensate for host-mediated or pharmacological MTOR suppression to facilitate maximal LCV expansion. Nevertheless, we provide evidence that *L*. *pneumophila* LCV expansion can be achieved through MTOR-independent mechanism(s) which function does not depend on the secretory pathway and are likely extensively utilized also by species other than *L*. *pneumophila*.

## Discussion

The building blocks of intracellular niches harboring pathogens are host-derived, thus the metabolic state of the host cell frequently dictates replication permissiveness [1,69,70]. As a central metabolic regulator in eukaryotes, MTOR is frequently targeted by viruses for metabolic reprogramming [71]. Here, we provide evidence that a bacterial pathogen subverts MTOR function to execute its niche biogenesis program.

Previously, we reported that in macrophage infections *Legionella* encoding a functional Dot/Icm system elicited a host-driven ubiquitination pathway that degrades activated Akt resulting in MTOR suppression and increased inflammation due to inefficient translation of anti-inflammatory mediators [51]. In this study, we provide evidence that host-driven MTOR suppression induced by *Legionella* is dependent on the TLR adaptor Myd88. The loss of host-mediated MTOR-suppression in *Myd88*^-/-^ BMMs revealed that *Legionella pneumophila* activates MTOR to reprogram infected cells metabolically for optimal expansion of its intracellular niche. The *Legionella* induction of MTOR required (i) a functional Dot/Icm apparatus; (ii) the Dot/Icm chaperone IcmS; and (iii) the host lipid kinase P3IK. These requirements are consistent with the existence of a *Legionella* Dot/Icm effector(s) that activates MTOR either directly by subverting PI3K or indirectly through LCV biogenesis. *Legionella* infections in the presence of cytochalasin D, a condition under which the Dot/Icm apparatus continues to translocate effectors but LCV formation is blocked, clearly demonstrates that the direct hypothesis holds true. The direct hypothesis is further supported by the observation that MTOR activation was specific to *L*. *pneumophila* and not several other *Legionella* species that encode diverse set of Dot/Icm effectors but establish similar ER-derived intracellular niches [72,73].

Our data indicate that the PI3K-Akt axis in *Legionella*-infected macrophages is subject to regulation by host-derived and bacteria-derived factors. PI3K signaling is induced by at least two regulators: (i) an activated TLR(s) [48,51] and (ii) a Dot/Icm effector protein; however, signaling is blocked downstream of PI3K by Myd88-dependent ubiquitination of Akt and MTOR [51]. This model reconciles the distinct MTOR signaling in *Legionella*-infected macrophages expressing or lacking Myd88. Our PI3K inhibition studies demonstrate that PI3K is required for *Legionella*-induced MTOR activation. In *Myd88*^-/-^ macrophages, the detection of sustained MTOR signaling argues that the host Akt/MTOR ubiquitination pathway requires priming by an activated TLR. When TLR function is intact, the degradation of activated Akt by the host ubiquitin ligases likely overrides the PI3K activation by the *Legionella* effector, thus suppressing the *Legionella*-driven MTOR signaling. Although the ubiquitin ligase for Akt has to be identified before this scenario can be tested experimentally, the loss of activated Akt is sufficient to reduce MTOR activation by TLRs through the PI3K/Akt axis [51] supporting the notion that MTOR induction by *Legionella* would be similarly suppressed. Thus, multiple signaling inputs impinge upon MTOR function in *Legionella*-infected cells.

Although sustained MTOR activation leads to significant pleiotropic reprogramming of cellular metabolism [39], our data demonstrates that *Legionella* benefits from the increase in host lipogenesis, which maximizes the housing capacity of the LCV likely by increasing membrane biogenesis. Eukaryotic membrane biogenesis requires constant supply of phospholipids and cholesterol that are derived by either *de novo* lipogenesis or by uptake from the extracellular environment [29]. In mammals, the circulatory system provides a rich and constant source of lipids to distal cells through the serum; In the presence of serum, cells favor lipids uptake over *de novo* lipogenesis to sustain membrane biogenesis because it is less energy expensive [29]. In our experiments we cultured terminally differentiated macrophages in serum-free cell culture conditions. In the absence of FBS, membrane biogenesis in infected BMMs requires *de novo* lipid synthesis because *Legionella* inhibits autophagy [74], a process that facilitates membranes/lipids recycling [75]. We provide evidence that when membrane biogenesis is dependent on *de novo* lipogenesis, LCV stability and expansion is mediated by the PI3K-MTOR-SREBP1/2 axis. Providing lipids exogenously reversed the LCV instability phenotype caused by PI3K or MTOR or SREBP1/2 inhibition, which is consistent with a LCV rupture caused by halted membrane biogenesis due to insufficient lipids supply.

MTOR can activate the lipogenic transcription factors SREBP1/2 via multiple mechanisms—increased mRNA stability, trafficking from the ER and interference with protein degradation in the nucleus [76]. MTOR activation by *Legionella* coincided with increased gene expression of *Srebf1* and its downstream target *Fasn and Ldlr* and preceded LCVs rupture indicating that at least partially SREBPs function in *Legionella*-infected cells is regulated through gene expression. However, post-transcriptional mechanisms might also contribute due to a global repression of protein synthesis that affects translation of some but not all polypeptides in infected cells [51,77,78]. Clearly, *Legionella* regulation of MTOR-dependent lipogenesis warrants future investigation.

Regardless of the precise mechanism, the importance of sustained MTOR activity for LCV homeostasis is evident by the premature loss of LCV integrity. Ruptured LCVs release microbial products in the host cytosol that induce flagellin-independent pyroptosis [18,26]. Similarly, we observed increased number of dead infected macrophages upon MTOR suppression either pharmacologically or genetically or by the host Akt/MTOR ubiquitination pathway. Importantly, the host cell death brought by MTOR inhibition required bacterial replication and was rescued when lipids were provided exogenously through serum or purified LDL particles further supporting the idea that MTOR activity regulates the lipid supply/demand ratio during the process of LCV expansion. Serum-derived lipids did not promote growth in axenic cultures nor in the early phase of intracellular replication, which supports a role for host-derived lipids in LCV biogenesis rather than bacterial replication and is consistent with the well documented *Legionella* utilization of carbohydrates and amino acids as biosynthesis and energy sources [3,79,80].

In mammalian infections, serum likely eliminates the *de novo* lipogenesis requirement for LCV homeostasis because inhibition of either pathway individually did not produce a LCV biogenesis defect. These data reconcile the lack of a cell death response in C57BL/6 macrophages infected with the *ΔflaA* strain in the presence of serum [52]. However, unicellular amoebae—the natural hosts for *Legionella—*inhabit aquatic environments in which lipids availability is likely similar to cell cultures under serum starvation conditions. In amoebae, such as *D*. *discoideum* and *A*. *castellani*, TORC1 metabolic functions are conserved [81], and in our studies TORIN2 moderately inhibited *L*. *pneumophila* intracellular replication in *A*. *castellani*. Because high MTOR activity overcomes premature lipids exhaustion during the late stages of LCV expansion it offers evolutionary advantage by increasing the number of bacteria released from infected cells. However, some large LCVs (>60 bacteria) were still detected upon lipid starvation thus a functionally redundant MTOR-independent pathway likely exits. In agreement, we also observed intracellular replication in an MTOR-independent manner by other *Legionella* species. Potentially, those species might subvert lipogenic factors downstream of MTOR or expand the LCV through fusion with membrane-bound vesicles or direct absorption of ER membranes. Disrupting the secretory pathway in the context of *L*. *pneumophila* infection did not exacerbate PP242-driven LCV instability in *Myd88*^*-/-*^ BMMs consistent with an important role of the secretory compartment in early LCV remodeling [6] but not late LCV expansion. Considering the knowledge gap in our understanding of the LCV expansion mechanisms it would be important to investigate the diverse expansion programs encoded by *Legionella* species.

MTOR subversion could be a strategy tailored for survival in a specific protozoan host that otherwise offers a moderate evolutionary advantage in other host species—a phenomenon discovered through en masse effector deletion studies [58]. Because of the critical link between membrane integrity of pathogen-occupied vacuoles and host defenses, MTOR subversion benefits would likely increase in hosts with robust restrictive mechanisms triggered by LCV instability and decrease in hosts that fail to restrict *Legionella* replication from leaky LCVs. Such a scenario is illustrated by the loss of the LCV stability regulator SdhA that impact intracellular replication but only in BMMs and not in *Dictyostelium* [26]. Thus, MTOR could be important for *L*. *pneumophila* survival in protozoan species that encode cytosolic macrophage-like defense responses.

However, differences in the wiring of MTOR circuits between mammalian and protozoan hosts and even between protozoa species could impact *Legionella* survival. For example, TORC2 regulates the TORC1 complex in macrophages but not *Dictyostelium* [81]. Also, *Legionella* uptake in macrophages but not *Dictyostelium* is PI3K-dependent [82,83]. In fact, loss of PI3K promotes *L*. *pneumophila* replication in *Dictyostelium* due to accumulation of phosphatidylinositol 4-phosphate, which facilitates early LCV remodeling, as well as decreased fusion with Nramp+ vesicles [82]. Conversely, in primary macrophages we observed increase in host cell death upon PI3K inhibition presumably due to LCV instability. The capacity of *Dictyostelium* protozoa to support robust replication of *L*. *pneumophila* residing in rupture-prone LCVs [26] could reconcile the phenotypic differences of PI3K inhibition during *L*. *pneumophila* infection.

Although our data established a requirement of continuous host lipids supply for LCV stability during expansion, we cannot differentiate between the need for a bulk increase in lipid content and the need for a specific lipid species on large LCVs. Little is known regarding the nature and dynamics of the LCV membrane lipid composition as the vacuole expands. Furthermore, *Legionella* secretes several lipid-modifying enzymes that potentially remodel the LCV membrane during infection [84,85]. As lipid content defines physical properties of biological membranes, enzymatic modifications could alter membrane tension leading to LCV rupture. Indeed, deletion of the secreted phospholipase PlaA stabilizes the rupture-prone vacuoles of the *ΔsdhA Legionella* [18]. In addition to SdhA, loss of several other Dot/Icm effectors with unknown functions results in a LCV instability phenotype [28,69]. The fact that *Legionella* has evolved multiple mechanisms to ensure LCV integrity, including metabolic reprogramming through MTOR, highlights the importance of niche homeostasis for *Legionella* intracellular survival.

Because MTOR represses autophagy, increased MTOR function could be an effective autophagy-suppressing mechanism encoded by *Legionella* that complements the LC3 protease RavZ [74]. Similar reactivation of MTOR by *Salmonella* suppresses autophagy induced by a host amino acids starvation response during infection of epithelial cells [50]. Under our experimental conditions, RavZ blocks autophagy regardless of MTOR activation state; thus, autophagy unlikely contributes to the cell death response elicited by MTOR suppression. Furthermore, rapamycin failed to disrupt LCVs and trigger the *Legionella*-induced cell death response despite a well-documented capacity to boost autophagy in BMMs [74]. Instead, our data indicates that LCV stability is regulated by a TORC1 rapamycin-insensitive or a TORC2 substrate. The mechanism of MTOR-driven lipogenesis in macrophages is poorly understood. Although in some cell types the TORC1 substrate S6K1 functionally links MTOR and SREBP1 [36], this is unlikely the case for *Legionella*-infected macrophages because S6K1 activity is sensitive to rapamycin. MTOR-dependent lipogenesis has exhibited both sensitivity and resistance to rapamycin depending on the cell type [36,60,61]. Because membrane biogenesis is critical for many physiological functions performed by macrophages, such as phagocytosis and protein secretion, it is important to dissect its mechanisms. In this aspect, *Legionella* infection could be an invaluable model to elucidate the mechanism of MTOR-induced lipogenesis.

For vacuolar bacterial pathogens, such as *Legionella*, host membranes are an integral part of niche homeostasis. Filling the large knowledge gap in our understanding of the niche biogenesis process is important for the development of not only anti-microbial agents but also for deciphering metabolic regulation. Our work establishes a mechanistic link between host lipids metabolism and *Legionella* intracellular niche homeostasis by defining a novel host determinant for LCV membrane integrity and setting the foundation for future research in the metabolism of host-pathogen interactions.

## Materials and Methods

### Ethics statement

All animal experiments were reviewed and approved by the Institutional Animal Care and Use Committee (IACUC) at the Louisiana State University Health Sciences Center-Shreveport. Approval notice (P-15-026).

### Bacterial strains

The following strains were used in this study: (1) *L*. *pneumophila* serogroup 1 strain JR32 deleted for *flaA* (Δ*flaA*) or *dotA* (Δ*dotA*) [52]; Lp02 pentuple strain [58]; (2) Lp02 thymidine auxotroph strains lacking *flaA* (*thyA* Δ*flaA*), *dotA* (*thyA ΔdotA*), *icmS* (*thyA* Δ*icmS*) [23] and *sdhA* (*thyA* Δ*sdhAΔflaA*) (this study); (3) Lp02 pentuple Δ*flaA* strain (this study); (4) *Legionella longbeachae* (ATCC 33462), *Legionella jordanis* BL-540 (ATCC 33623) and *Legionella dumoffii* (ATCC 33279) species were kind gift from Dr. Nicholas Cianciotto (Northwestern University). *Legionella* strains were grown on charcoal yeast extract (CYE) plates [1% yeast extract, 1%*N*-(2-acetamido)-2-aminoethanesulphonic acid (ACES; pH 6.9), 3.3 mM l-cysteine, 0.33 mM Fe(NO3)_3_, 1.5% bacto-agar, 0.2% activated charcoal] [86]. Lp02 derived strains were grown on CYE plates containing thymidine at 100μg/ml. For all experiments, *Legionella* were harvested from CYE plates after growth for 2 days at 37°C.

### Plasmids and strain construction

The Δ*sdhA* and the Δ*flaA* allele was generated by recombinant PCR using a ~1kb region upstream of the gene using the following primers for Δ*sdhA* (forward primer 5’-CGGGATCCCCTGATATAATGAAAAATATAGCCC-3’ and reverse primer 5’-CGCGGCCGCATTTCAATATTAAAAAAATTAACTTGTG-3’), a downstream region of *sdhA* (forward primer 5’-CGCGGCCGTAAAAATATTTGATTTAATCTTTTAATTTACC-3’ and reverse primer 5’-GCGAGCTCTGTCCATTTTTTCATACGAAAATACC-3’) and for Δ*flaA* (forward primer 5’-CGGGATCCTTCGTTGAAAGCCTTCTGGC-3’ and reverse primer 5’-CCGCTCGAGGGATGTCGCAATCGAAGTGC-3’), a downstream region of *ΔflaA* (forward primer 5’-CCGCTCGAGTCTCCTCAGACCTGAATCC-3’ and reverse primer 5’-AAACTGCAGCAGTTAATGAATTCACTCCC-3’). The resulting fragments were linked by a EagI site for Δ*sdhA* and XhoI site for Δ*flaA* and cloned into the BamHI/SacI sites of the gene replacement vector pSR47s creating pSR47s-CD0376 and pSR47s-CD1340 respectively. Strain Lp02 Δ*flaA* Δ*sdhA* was derived from LP02 Δ*flaA* and generated by allelic exchange of *sdhA* with Δ*sdhA* and the Lp02 pentuple Δ*flaA* was derived from LP02 pentuple and generated by allelic exchange of *flaA* with Δ*flaA*. The allelic exchange was confirmed by genotyping clonal isolates with the following primers in a single PCR reaction for *sdhA* (5’-CATTGAGCGTATCAAACACG-3’, 5’-GTAAAATGATAATTGAAGAGG-3’ and 5’-CAGTCTGGGGTTGAAATGTCC-3’) or for flaA (5’-TTGTGCAGTCATTCGTTGAG-3’, 5’-GTATTGATTATATTACAATCG-3’ and 5’-TCATCAGAAGGCATTTTACG-3’) that produces a fragment of ~330bp for *sdhA/flaA* or a fragment of ~500bp for Δ*sdhA/ΔflaA*.

### Reagents

The pharmacological agents used in the study were obtained from the following suppliers Cell Signaling (LY294002), Sigma (PP242), Biolegend (Brefeldin A), Cayman Chemical Company (Torin2, Cytochalasin D and Nocodazole), CalBiochem (InSolutionRapamycin) and R&D Systems (Fatostatin). Primary antibodies were purchased from Cell Signaling– α-Akt(pan) (C67E7), α-rS6p(5G10), α-prS6p(S235/236)(D57.2.2E) and α-pS6K1(T389)(108D12); Enzo Life Sciences—α-Ubiquitinated proteins (FK2); Abcam– α-Galectin-3 (A3A12); Santa Cruz—α-Actin (C-2). The following antibodies were custom produced by Cocalico Biologicals against formalin-killed bacterial strains– α-*L*. *pneumophila* (chicken IgY), α-*L*. *dumoffii* (chicken IgY), α-*L*. *longbeachae* (rabbit IgG) and α-*L*. *jordanis* (rabbit IgG). Secondary antibodies and Hoechst 33342 (cat #H3570) were purchased from ThermoFisher Scientific—α-mouse-Alexa488 (cat #A11001), α-mouse-Alexa647 (cat #A21235), α-rabbit-Dylight550 (cat #SA5-10039), α-rabbit-Alexa647 (cat #A21244), α-chicken-IgY-FITC (cat #A16055), α-chicken-IgY-TRITC (cat #A16059). Complete FBS was purchased from Seradigm (cat #1300_500). For the studies with delipidated-serum, FBS was purchased from Gemini Bio-products: complete FBS (cat #100–106), delipidated FBS (cat #900–123). The cholesterol and triglycerides content of FBS was verified using biochemical assays. Purified human LDL was purchased from Intracel (cat #RP-032, Intracel Frederick, MD).

### Mice

For these studies bone-marrow was harvested from C57BL/6J (Jackson Labs, cat # 000664), *Myd88*^-/-^ (Jackson Labs, cat #009088) and mTOR^fl/fl^Cre+ mice (*Mtor*^-/-^). Mice with *Mtor* deletion in the myeloid compartment were generated by breeding mTOR^fl/fl^ (Jackson Labs, cat # 011009) harboring *loxP* sites flanking exons 1–5 of the *Mtor* locus with LyzM^Pro^ Cre+ transgenic mice (Jackson Labs, cat #004781) [51].

### BMM and U937 derivation and cell culture

To derive BMMs, bone marrow cells were isolated from the femurs and tibiae of mice, and cultured in RPMI 1640 with L-glutamine (Sigma, cat #R8758) containing 10% FBS (Seradigm), 20% macrophage colony-stimulating factor (M-CSF)-conditioned medium, and 1% primocin (Invivogen) at 37°C with 5% CO_2_. Equal volume of media was added on day 4. On day 7, cells were harvested and used for infection assays. M-CSF condition media was obtained from an L-929 fibroblast cell line (ATCC). U937 cells (ATCC) were cultured in RPMI 1640 containing 10% FBS (Seradigm) and 1% Pen-Strep (ThermoFicher) at 37°C with 5% CO_2_ and were differentiated with 10ng/ml Phorbol 12-myristate 13-acetate (Adipogen) for 24hrs followed by culture in PMA-free media for 48hrs prior to infections.

### Infections with *Legionella* strains for immunofluorescence analysis

For infections, BMMs were seed on cover slips in re-plating media (RPMI 1640 with L-glutamine, 10%FBS, 10% M-CSF-conditioned media) for 8 hrs prior to serum starvation. U937 were seeded in 24-well plates and PMA-differentiated directly onto cover slips. For all experiments cell were serum starved for 10 hrs by culturing in RPMI 1640 with L-glutamine (serum-free RPMI). BMMs (2.5x10^5^ per well) or PMA-differentiated U937 (5x10^5^ per well) seeded on cover slips in 24-well plates were infected with *Legionella* at MOIs as indicated for each experiment. At 60 min post infections extracellular bacteria were removed by washing 5X with warm (37°C) phosphate-buffered saline (PBS) and the infected BMMs were cultured in serum-free RPMI. Inhibitors and FBS or dFBS were added as indicated for each experiment. The duration of infection is noted for each experiment.

For immunostaining, coverslips containing cells were washed with 3x PBS and fixed with 2% paraformaldehyde (PFA) for 20 min, permeabilized with cold methanol for 30 seconds, blocked with 2% BSA in PBS for 60 min. Primary antibodies dilutions were 1:500 for α-p-rS6p, 1:200 for α-ubiquitin (FK2), 1:200 for α-Galectin 3 and 1:1000 for all α-*Legionella* antibodies. Primary antibodies were incubated in PBS containing 1% BSA overnight at 4°C. Secondary antibodies were used at 1:500 dilution and Hoechst 33342 at 1:2000 for 60 min at room temperature. Coverslips were mounted with ProLong Gold antifade reagent (ThermoFisher) onto slides and examined by fluorescence microscopy. For detection of leaky LCVs by selective permeabilization cell were fixed with 2% PFA for 20min, incubated with α-ubiquitin (FK2) and α-*L*. *pneumophila* chicken ab in PBS with 0.1% saponin and 2% BSA overnight at 4C. Next, coverslips were extensively washed with PBS, incubated with cold 100% methanol for 30 sec, extensively washed with PBS and incubated with α-*L*. *pneumophila* rabbit ab for 2hrs. Secondary antibodies were applied as described above.

### *Legionella* intracellular replication in *Acanthamoeba castellanii*

*A*. *castellanii* (ATCC) was propagated in ATCC Medium 712: PYG w/ Additives at room temperature. For infections, amoebae were seeded at 5X10^5^ cells per well in a 24 well tissue culture plates in A.c. buffer and allowed to settle down for 60 min. Next, amoeba were infected with JR32ΔflaA *Legionella* in A.c. buffer at MOI = 5 in the presence of DMSO or Torin2 (300nM) in technical triplicates for each condition. Inhibitors were added at the time of infection. At 60min post infection and at 48hrs post-infection, cells were collected and mechanically disrupted by passaging 10 times through a 27G syringe needle and various dilution were plated on CYE agar plates.

### Microscopy analyses of infected cells

3D images were acquired with inverted wide-field microscope (Nikon Eclipse T*i*) controlled by NES Elements v4.3 imaging software (Nikon) using a 60X/1.40 oil objective (Nikon Plan Apo λ), LED illumination (Lumencor) and CoolSNAP MYO CCD camera. Image acquisition, deconvolution and analysis was completed with NES Elements v4.3 imaging software. Only linear image corrections in brightness or contrast were completed. For all analyses, three-dimensional images of randomly selected fields were acquired and image acquisition parameters were kept constant for all the cover slips from the same experiment.

**For phospho-rS6p analysis in cells**, the ubiquitin signal was utilized to define a binary mask for each cell from which the mean fluorescence intensity of p-rS6p was obtained. Background fluorescence signal from each acquired field was individually determined from a cell-size mask positioned in an unoccupied area and subsequently subtracted from the MFI of each cell in the field.
**For imaging Galectin 3 LCV recruitment**, each plane of the Z-stack was scrutinized to ensure that multiple Galectin 3 puncta localize proximal or co-localized with the bacteria. Because Galectin 3 binds exposed sugar moieties on ruptured membranes, LCVs were considered positive if the galactin 3 wrapped the bacteria or greater than 4 puncta were proximal to the bacteria (< 0.5μm).
**For cell death analysis**, we set binary mask using Hoechst fluorescence to define and measure individual objects larger than 5μm^3^. Cell with aberrant nuclear morphology were obvious even by manual scoring and were marked by decrease in nuclear volume and increase in Hoechst 33342 fluorescence.
**For LCV size analysis**, 3D images of infected cells stained with purified anti-*Legionella* IgY chicken antibody were acquired by setting the Z-distance between the immediate out-of-focus planes to capture the whole cell. The space between imaged planes was set at 0.3μm, thus depending on the cell type the number of planes in a given Z-stack varied. 3D binary masks were set for each field of cells individually based on bacterial fluorescence to define objects and to ensure out of focus signal was omitted from the mask. Due to the high fidelity of the anti-*Legionella* IgY antibody, images were not deconvoluted prior to binary mask definition because deconvolution did not improve data acquisition. The volume of each object was measured with the 3D image volume analysis function of NES Elements (Nikon). To derive the mathematical algorithm for volume-to-bacteria conversion the object volumes of LCVs containing bacteria that can be definitively manually enumerated were determined and plotted against the data from the bacterial count per vacuole. The best linear fit algorithm for the plot was used to derive the conversion equation (Y = 0.5842*X+0.4835, X = object volume [μm^3^] Y = number of bacteria). The conversion algorithm was back tested by extrapolating bacterial numbers of LCVs in which the number of bacteria can be manually counted definitively. (see S8 Fig)

### Infections with *Legionella* strains for LDH release assay

BMMs were seeded in 48-well plates (1X10^5^ cells per well), serum-starved for 10 hrs and infected as indicated at MOI = 10 in technical triplicates for each time point. Total supernatant volume was 200μl. For the FBS rescue experiments, the serum was added at the time of infection. Supernatants were collected and immediately frozen at -20C. Thawed supernatants (50μl) were assayed for LDH activity using the CytoTox 96 Non-Radioactive Cytotoxicity Assay (Promega) according to the manufacturer’s protocol. Supernatants from media only, unifected cell and Triton X-100 lysed cells were used to calculate the dynamic range of the assay as per the manufacturer’s instructions.

### Infections with *Legionella* strains for immunoblot analysis

BMMs were seeded in 12-well plates (8X10^5^ cells per well), serum-starved for 10 hrs and infected as indicated. Cells were washed with cold PBS and lysed with RIPA buffer (20mM Tris-HCl pH7.5, 200mM NaCl, 1mM EDTA, 1% NP-40, 1% sodium deoxycholate, 2.5mM sodium pyrophosphate, 1mM β-glycerophosphate, 1mM Na_3_VO_4_, 1μg/ml leupeptin). Total cell lysates (20μg) were resolved by SDS-PAGE, transferred onto nitrocellulose membranes blocked with 2% BSA and incubated with primary antibody for either 3 hrs at room temperature or overnight at 4°C for phospho-antibodies. Primary antibody dilutions were used as per manufacturers’ instructions. Secondary horseradish peroxidase-conjugated antibodies (ThermoFisher) were used at 1:5000 in 2 hrs incubations at room temperature. Signals were visualized with ECL (GE Healthcare) and analyzed with ImageJ (NIH).

### Quantitative PCR analysis of gene expression

*Myd88*^-/-^ BMMs (1X10^6^ cells) were serum starved and infected under serum-free conditions as indicated for 6 hrs. Total RNA and first-strand cDNA synthesis was performed with TaqMan Gene Expression Cells-To-Ct Kit (ThermoFisher) according to the manufacturer’s instructions. mRNA levels were determined by quantitative real-time PCR using the Universal ProbeLibrary (Roche, Life Science) and LightCycler 480 Probes Master (Roche, Life Science). Thermal cycling was carried out using a LightCycler 96 instrument (Roche Diagnostics) under the following conditions: 95°C for 5 min and 45 cycles at 95°C for 10 sec and 60°C for 25 sec. Gene expression was normalized to *Canx* (Calnexin). The fold increase is shown relative to uninfected cells (value of 1).

### Statistical analysis

Calculations for statistical differences were completed by Student’s T-test or ANOVA as indicated using Prism v6 (GraphPad Software).

## Supporting Information

S1 Fig Microscopy analysis of rS6p phosphorylation in macrophages infected by *Legionella*. **(a-b)** Images from synchronized infections under serum-free conditions of serum-starved BMMs infected as indicated. Representative single projection images of 3D microscopy images of macrophages infected by Δ*flaA* **(a)** or Δ*dotA* **(b)** for 8 hrs (MOI = 20, infections synchronized at 60min) and stained with anti-phospho-rS6p(S235/S236), anti-*Legionella* (L.p) antibodies and Hoechst 33342. (*) indicates *Legionella* infected cell, Bar = 10μm. **(c-d)** Frequency distribution of the phospho-rS6p mean fluorescent intensity (MFI) in individual macrophages infected by Δ*flaA* **(c)** or Δ*dotA* **(d)**. Each MFI bin spans 200 fluorescence intensity units of the p-rS6p(S235/S236) fluorescent signal. At least 200 LCVs were analyzed for each condition. A representative of three **(a-d)** biological replicates is shown for each experiment.
(EPS)

S2 Fig Analysis of rS6p phosphorylation in *Myd88*^-/-^ BMMs co-infections with different *Legionella* species. **(a-b)** Images from synchronized infections under serum-free conditions of serum-starved *Myd88*^-/-^ BMMs for 8 hrs (MOI = 20) as indicated. Cells were infected by either *L*.*pneumophila* (LP), *L*.*dumoffii* (LD), *L*.*longbeachae* (LL) or the indicated combinations in the co-infection (ratio 1:3) experiments. The Δ*flaA* of *L*.*pneumophila* was used to avoid pyroptosis. LD and LL lack the *flaA* gene [87]. Quantitated data is shown in **(a). (b)** A representative micrograph of cells stained with anti-ubiquitinated proteins (FK2), anti-p-rS6p (S235/236), anti-*L*.*dumoffii* antibodies and Hoechst 33342. Each channel is shown individually. Ubiquitin is a marker specific for LP LCVs and was used to identify LP-infected macrophages. Arrowheads indicate *Legionella*-containing vacuoles and (*) mark cells co-infected by LP and LD, Bar = 10μm. **(c)** Quantitation of p-rS6p positive *Myd88*^*-/-*^ BMMs uninfected or infected with either *ΔflaA* or pentuple *ΔflaA* strains for 8hrs. Synchronized infections with MOI = 20 **(a, c)** Means ± s.d of technical triplicates for the two distinct groups within the cell population–**(a)** LP present and LP absent for each condition are shown. At least 50 infected cells were analyzed for each condition. A representative of two biological replicates is shown. **(a** and **c)** n.s—not significant (unpaired T-test).
(EPS)

S3 Fig Microscopy analysis of infected BMMs with aberrant nuclear morphology. **(a-b)** Panel of micrographs of representative Δ*flaA* infected BMMs stained with anti-*L*. *pneumophila* antibody and Hoechst 33342. Cells with aberrant **(a)** or normal **(b)** nuclear morphology are shown. Bar = 10μm.
(EPS)

S4 Fig Quantitative PCR analysis of gene expression in *Myd88*^-/-^ BMMs infected with *Legionella*. **(a)** qPCR analysis of gene transcripts that regulate lipogenesis and lipids trafficking in serum-starved BMMs infected by either Δ*flaA* or Δ*dotA* (MOI = 10) for 6 hrs under serum-free conditions. Means ± s.d of technical triplicates for each condition are shown. The amount of each transcript was normalized to *Canx* (Calnexin) and is presented as fold increase over unstimulated cells (UN). A representative of two biological replicates is shown. n.s—not significant, * p<0.05, ** p<0.005 (unpaired T-test).
(EPS)

S5 Fig Exogenous lipids rescue the cell death response to *Legionella* infection brought by MTOR-suppression in human U937 macrophages. **(a-b)** Phorbol ester differentiated U937 cells were serum-starved and infected under serum-free conditions with Δ*flaA Legionella* for 16 hrs (MOI = 10, synchronized infections) in the presence/absence of PP242 (2.5μM). **(a)** Micrographs show representative populations of macrophages stained with anti-*L*. *pneumophila* (L.p) and Hoechst 33342. Arrowheads indicate LCVs and (*) indicate infected cells with condensed nucleus. (Bar = 10μm.) **(b)** Quantitation of infected and neighboring uninfected U937 macrophages with condensed nuclei after the indicated treatments. Means ± s.d of technical replicates of dead cell as percentage of total cells in each condition are shown. A representative of two biological replicates is shown. n.s—not significant, ** p<0.005 (unpaired T-test).
(EPS)

S6 Fig Galectin 3 recruitment to *Legionella* infected BMMs. **(a-c)** Serum-starved *Myd88*^-/-^ macrophages were infected with Δ*flaA L*. *pneumophila* for 10 hrs **(a-b)** or *L*. *dumoffii* for 12 hrs **(c-d)** in synchronized infections in the absence **(a-d)** or presence **(d)** of FBS (10% v/v). **(a-c)** Micrographs of representative Galectin 3 negative **(a)** and positive **(b** and **c)** vacuoles are shown. Cells were stained with anti-galectin3, Hoechst 33342 and anti-*L*. *dumoffii* (L.d) or anti-*L*. *pneumophila* (L.p) antibodies. Arrowheads indicate the LCVs. **(a)** Cell harboring Galectin 3 negative LCVs show typical dispersed Galectin 3 staining pattern, whereas cell containing Galectin 3 positive LCVs show distinct accumulation of multiple bacteria-proximal Galectin 3 puncta **(b** and **c)**. **(d)** Quantitation of Galectin 3-positive LCVs produced by infections with Δ*flaA* or *L*.*dumoffii* and treatments with PP242 (2.5μM) or vehicle alone. Means ± s.d of technical triplicates for each condition are shown. At least 100 LCVs were analyzed for each condition. A representative of two biological replicates is shown. n.s—not significant, ** p<0.005 (unpaired T-test).
(EPS)

S7 Fig Fatostatin, PP242 and Torin2 do not interfere with *Legionella* growth in axenic cultures. **(a-b)** *Legionella* growth in AYE liquid cultures in the presence/absence of fatostatin (40μM) **(a)**, PP242 (25μM) **(b)**, Torin2 (3μM) **(b)**. Changes in the cultures’ optical densities over time are shown for each condition. A representative of two biological replicates are shown for each experiment.
(EPS)

S8 Fig Three dimensional microscopy analysis of the number of bacteria per LCV in infected cells. **(a)** Methodological outline for the analysis of LCV bacterial size from 3D microscopy images of infected cells stained with purified anti-*Legionella* IgY chicken antibody. Z-stack series of a single LCV spanning 2.4 μm and containing four bacteria is shown. Each bacterium is numbered and the outline of the 3D binary mask used to measure the LCV volume (5.65 μm^3^) is shown. **(b)** Derivation of the LCV volume-to-bacteria number conversion formula. Graph plots the measured volumes of the bacterial mass for 100 LCVs in which the individual bacteria can be unambiguously identified over the manually counted number of bacteria per LCV. **(c)** Micrographs of image projections of individual representative LCVs used in the formula derivation protocol. Each bacterium is numbered and the volume of the bacterial mass binary mask is shown. The volume of individual bacteria varied from 0.95μm^3^ to 2.42μm^3^. A bacterium undergoing binary fission was counted as a single cell until the septum is resolved. **(d)** Back-testing of the conversion formula derived in **(b)**. The number of bacteria per LCV (counted manually vs. calculated from volume measurements) is plotted for over 100 LCVs. Circles in red color denote perfect correlation. Circle size in the graph positively correlates with the number of LCVs that were assigned to each circle. **(e)** Z-stack series of a single large LCV from a U937 macrophage infected for 16hrs. The outline of the binary mask used to generate the volume measurement is shown for each 0.3μm Z-slice. The LCV volume (211μm^3^) was used to calculate the size (124 bacteria) using the formula derived in **(b)**. The LCV Z-stack series is animated in the S1 Video. **(f)** Projection micrograph of the macrophage containing the LCV analyzed in **(e)** was stained with anti-ubiquitinated proteins (FK2), anti-*Legionella* antibodies (L.p) and Hoechst 33342.
(EPS)

S1 Video Three dimensional microscopy analysis of the number of bacteria per LCV in infected cells. Three-dimensional animation of the large LCV shown in S8f Fig with the edges of the binary mask used for volume analysis highlighted in green.
(AVI)
